# Cardiovascular events among patients with prostate cancer treated with abiraterone and enzalutamide

**DOI:** 10.2340/1651-226X.2024.20337

**Published:** 2024-04-09

**Authors:** Onur Baser, Gabriela Samayoa, Archana Dwivedi, Sara AlSaleh, Burhan Cigdem, Erdi Kizilkaya

**Affiliations:** a Department of Economics, Bogazici University, Bebek, Istanbul, Turkiye; b Department of Internal Medicine, University of Michigan, Ann Arbor, MI, USA; c Graduate School of Public Health, City University of New York, New York, USA; d Columbia Data Analytics, New York, New York, USA; e Columbia Data Analytics, Ann Arbor, Michigan, USA; f Mergen Analytics, Ankara, Turkey

**Keywords:** Prostate cancer, abiraterone, enzalutamide, cardiovascular adverse events, androgen deprivation therapy, hormone therapy

## Abstract

**Background and purpose:**

There is growing concern about the adverse metabolic and cardiovascular effects of abiraterone acetate (AA) and enzalutamide (ENZ), two standard hormonal therapies for prostate cancer. We analysed the risk of cardiovascular adverse events among patients treated with AA and ENZ.

**Patients and methods:**

We used Kythera Medicare data from January 2019 to June 2023 to identify patients with at least one pharmacy claim for AA or ENZ. The index date was the first prescription claim date. Patients were required to have 1 year of data pre- and post-index date. New users excluded those with prior AA or ENZ claims and pre-existing cardiovascular comorbidities. Demographic and clinical variables, including age, socioeconomic status (SES), comorbidity score, prostate-specific comorbidities, and healthcare costs, were analysed. Propensity score matching was employed for risk adjustment.

**Results:**

Of the 8,929 and 8,624 patients in the AA and ENZ cohorts, respectively, 7,647 were matched after adjusting for age, sociodemographic, and clinical factors. Between the matched cohorts (15.54% vs. 14.83%, *p <* 0.05), there were no statistically significant differences in any cardiovascular event after adjusting for these factors. The most common cardiovascular event in both cohorts was heart failure (5.20% vs. 4.49%), followed by atrial fibrillation (4.42% vs. 3.60%) and hypotension (2.93% vs. 2.48%).

**Interpretation:**

This study provides real-world evidence of the cardiovascular risk of AA and ENZ that may not appear in clinical trial settings. Adjusting for age, baseline comorbidities, and SES, the likelihood of a cardiovascular event did not differ between treatment groups.

## Introduction

Prostate cancer is the second most common cancer in the United States of America (US), comprising 9.5% of all new cancer cases recorded in 2018 [1], and approximately 34,500 deaths each year [2]. It is the sixth-leading cause of cancer mortality in men worldwide [3]. Metastatic castration-resistant prostate cancer (mCRPC) is the most frequent cause of prostate cancer–related death [3]. Men who progress to mCRPC have a poor prognosis, with a median overall survival of 25.6 months [2, 4–6].

Androgen deprivation therapy (ADT) is central to treating locally advanced and metastatic disease [7] by blocking the production of testosterone or curtailing its function to stop prostate cancer growth. Abiraterone acetate (AA), an androgen biosynthesis inhibitor, and enzalutamide (ENZ), an androgen receptor signalling inhibitor, are standard hormonal therapies that are mainstay additions to ADT. Both AA and ENZ have been approved for use in pre-chemotherapy and post-chemotherapy settings, demonstrating satisfactory efficacy and tolerability [3]. Both AA and ENZ have been proven to increase the survival of patients with CRPC and, more recently, of patients with metastatic hormone-sensitive disease naive to hormonal agents [8, 9]. However, adverse drug effects are common with these hormonal therapies and may vary with patient and drug characteristics [10, 11]. Furthermore, cardiovascular disease (CVD) is a primary cause of noncancer mortality in men with prostate cancer [12]. A recent study identified that CVD accounted for almost 30.2% of all deaths among prostate cancer patients [13]. Thus, there is a growing concern about the adverse metabolic and cardiovascular effects [14] of ADT due to the higher risk of CVD associated with the therapy in a population susceptible to CVD.

Real-world studies showing the association between cardiovascular events related to hormonal treatments and pre-existing metabolic, cardiovascular, or neurological conditions are limited [7, 10, 15–17]. Furthermore, because differential adverse effects of AA and ENZ and their interactions with patient comorbidities have not been fully elucidated, there is little guidance on how to choose these drugs based on pre-existing conditions [10].

This retrospective cohort study aims to investigate the likelihood of experiencing negative cardiovascular events during treatment with AA and ENZ. The study also intends to provide useful insights that can assist physicians in making informed decisions about patient care.

## Patients and methods

We conducted a retrospective cohort study using Kythera Medicare data from January 2019 to June 2023. Kythera Labs’ data contains medical and pharmacy claims with 79% coverage of all US patients. In addition to commercial and Medicaid plans, the data include both Medicare Fee-for-Service and managed-care patients. Out of 65 million Medicare enrolees, the data contains 58 million random patients. Overall, the data covers approximately 275 million patients, 3 million practitioners, 400,000 organisations, and 1.2 million facilities, generating 9.7 billion healthcare claims. Data include the unique de-identified numbers of patients, age, gender, types of insurance (fee-for-service vs. managed care), zip codes, diagnosis according to the *International Classification of Diseases* (ICD-10), *Current Procedural Terminology* (CPT) codes, and National Drug Codes (NDC) for medications. Since each patient is allocated, a unique identifier links all encounters, allowing for longitudinal analysis. The details of the data have been published elsewhere, and the healthcare outcomes derived from these data were compared with other data sets for their validity and consistency [18].

**Figure 1:**
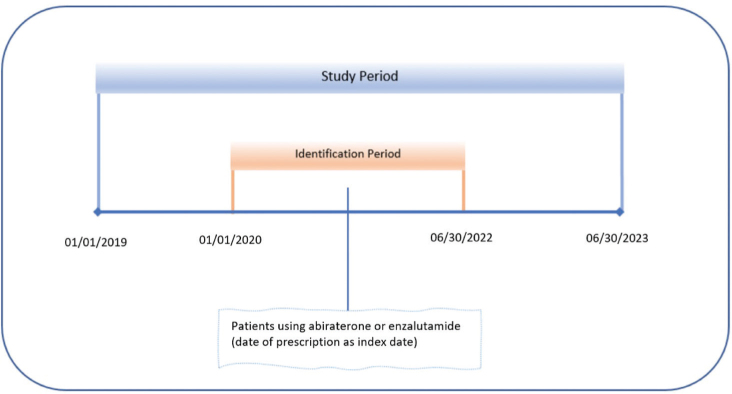
Study timeline.

We identified patients with prostate cancer who had at least 1 pharmacy claim for AA or ENZ during the identification period. The first prescription claim date was considered the index date. Patients were required to be in the data at least 1 year pre- and post-index date. Prostate cancer was identified using the appropriate ICD-10 code (ICD 10 C61, Z85.46). Patients with at least 1 claim with a diagnosis of prostate cancer during the baseline are included in the study (Figure 1).

To identify new users, we excluded patients if they had a claim of AA or ENZ prior to the identification period. To distinguish new cases of CVD from ongoing episodes, we excluded patients with cardiovascular comorbidities prior to the index date. Patients with a claim for AA and ENZ at the same time on the index date were also excluded. We analysed a set of demographic and clinical variables at the baseline and during the follow-up period. Age was determined from the relevant file in the data at the index date.

We constructed a summary measure of socioeconomic status (SES) for each US zip code using data on income, education, and occupation from the 5-year estimates for 2021 US Census data [19]. Five-year estimates refer to statistical projections released by the Census Bureau every 5 years, covering various geographic, demographic, and socioeconomic factors. We then linked this information to the enrolees’ zip code of residence in the Kythera files. Previous research identified six variables by factor analysis of census block groups that could be meaningfully combined into a summary SES score. These variables include three measures of wealth/income (log of the median household income, log of the median value of housing units [20], and the percentage of households receiving interest, dividend, or net rental income [21], two measures of education (the percentage of adults ≥ 25 years of age who had completed high school or the percentage of adults ≥ 25 years of age who had completed college) [22], and one measure of occupation/employment (the percentage of employed persons ≥ 16 years of age in executive, managerial, or professional specialty occupations) [22]. The *z* score for each variable was calculated by subtracting the overall mean and dividing it by the standard deviation. The SES score was then constructed by summing the *z* scores for each of the six variables.

Additionally, different clinical measures were derived: updated Charlson Comorbidity Index (CCI), Chronic Disease Score (CDS), and Elixhauser Index. The original CCI encompassed 19 categories of identifiable medical conditions [23]. The original index, which has since adopted several weights, some of which allow outpatient diagnoses to contribute to the score, was translated to the updated ICD-10 codes [23]. The CDS is an aggregate comorbidity measure based on current medication use [24]. The score increases with the number of chronic diseases under treatment and the complexity of the treatment regimen. The Elixhauser Index is based on a comprehensive set of 30 ICD-9-CM comorbidity flags and has been updated to ICD-10 codes [25]. Current coding for the index is available from the Agency for Healthcare Research and Quality. It has been shown that using all these indexes as a proxy of disease severity improves the performance of the outcomes research models [26].

We identified prostate-specific comorbidities (depression, diabetes, gastric acid disorders, hyperlipidemia, osteoporosis, asthma, chronic obstructive pulmonary disease, Alzheimer’s disease, and chronic pain syndrome) [27] and baseline total healthcare cost to proxy for the severity of prostate cancer. The incidence of a cardiovascular event was defined as presence of hypertension, ischemic heart disease, myocardial infarction, heart failure, ventricular arrhythmias, cerebrovascular accidents, peripheral vascular disease, atrial fibrillation, paroxysmal tachycardia, cardiomyopathy, hypotension, pulmonary embolism, atherosclerosis, or aortic aneurysm during the year after initiation of index medication.

Baseline and outcome variables were analysed descriptively. Numbers and percentages were provided for categorical variables; means and standard deviations were provided for continuous variables. Student’s *t*-tests and Pearson chi-squared tests were used to test statistically significant differences at the 5% level for continuous and categorical variables, respectively. Standardised differences (STDs) were calculated to distinguish practical from statistical significance, and STDs with a value greater than 0.01 were considered significant.

To control the non-random assignment of patients, we constructed logistic regression models that predicted the likelihood of using each medication (the propensity score) and matched patients in each cohort by this score. We used as explanatory variables all demographics (age, SES), clinical characteristics (CCI, CDS, and Elixhauser Index scores), baseline prostate-specific comorbidities, and healthcare costs at baseline. McNemar’s test for categorical variables or a paired Student’s *t*-test for continuous variables was used to account for the dependence of matched pairs. All analyses were conducted using Pyspark and SparkR on Databricks.

## Results

Among the initially identified 26,035 patients in the AA cohort and 24,921 patients in the ENZ cohort, a total of 8,929 patients met the criteria for AA treatment, resulting in a retention rate of approximately 34.28% from the initial cohort. Within the ENZ group, 8,624 patients qualified for treatment, with a retention rate of approximately 34.58% from the initial cohort. The mean patient age was 71.58 years in the AA group and 73.40 years in the ENZ group (*p* < 0.05). The analysis of comorbidity scores revealed notable differences between the groups. Specifically, both the CCI score (2.19 vs. 2.28), and the Elixhauser score (3.29 vs. 3.43) were significantly higher (*p* < 0.05) in the ENZ cohort, while the CDS showed no statistically significant difference.

Our results indicated that patients who reside in high-SES score regions were more likely to use AA than ENZ (35.39% vs. 30.61%, *p* < 0.05). On the contrary, patients who reside in low-SES score regions were more likely to use ENZ than AA (29.15% vs. 35.12%, *p* < 0.05).

Table 1 compares the baseline characteristics and prevalence of various prostate cancer–specific comorbidities in the treatment groups. Diabetes (17.38% vs. 23.12% was the most prevalent comorbidity among the ENZ group (*p* < 0.05) and hyperlipidemia (21.02% vs. 21.93%) was the most common comorbidity among the AA group (not significant). Additionally, depression (5.62% vs. 4.87%) and asthma (2.70% vs. 2.18%) were significantly more common in the AA group than in the ENZ cohort (*p* < 0.05). Although gastric acid disorders were more common in the AA cohort, and chronic obstructive pulmonary disease, osteoporosis, pain syndrome, and Alzheimer’s disease were more prevalent among the ENZ cohort, these differences were not statistically significant. Total baseline health expenditure, however, was slightly higher for the AA group ($25451.16 vs. $21540.64, *p* < 0.05).

**Table 1:** Baseline characteristics of the study and comparison cohorts.

Variables	Abiraterone (N = 8,929)		Enzalutamide (N = 8,624)		*p*	STD
	N/Mean	%/STD	N/Mean	%/STD		
Age (years)	71.58	9.12	73.40	9.01	< 0.0001	0.2013
Age group						
65–74	3566	39.94	3182	36.90	< 0.0001	0.0625
75–84	2491	27.90	2803	32.50	< 0.0001	0.1004
85+	867	9.71	1168	13.54	< 0.0001	0.1200
Comorbidity score						
CCI	2.19	1.11	2.28	1.18	< 0.0001	0.0793
CDS	2.23	2.58	2.21	2.63	0.6311	0.0072
Elixhauser Index	3.29	2.03	3.43	2.12	< 0.0001	0.0699
SES score by tertile						
Low	2603	29.15	3029	35.12	< 0.0001	0.1282
Medium	2915	32.65	2718	31.52	0.1090	0.0242
High	3160	35.39	2640	30.61	<0.0001	0.1017
Comorbidities						
Depression	502	5.62	420	4.87	0.256	0.0337
Diabetes	1552	17.38	1994	23.12	< 0.0001	0.1433
Gastric acid disorders	896	10.03	841	9.75	0.5304	0.0095
Hyperlipidemia	1877	21.02	1891	21.93	0.1440	0.0221
Osteoporosis	376	4.21	372	4.31	0.7367	0.0051
Asthma	241	2.70	188	2.18	0.0260	0.0336
COPD	458	5.13	484	5.61	0.1558	0.0214
Alzheimer’s disease	45	0.50	49	0.57	0.5601	0.0088
Pain syndrome	107	1.20	115	1.33	0.4231	0.0121
Health expenditures						
Total cost	$25,451.16	$29,989.31	$21,540.64	$25,258.94	<.0001	0.1408

CCI: Charlson Comorbidity Index; CDS: Chronic Disease Score; COPD: chronic obstructive pulmonary disease; SES: socioeconomic status; STD: standardised difference.

Table 2 describes the incidence of cardiovascular events after initiation of AA or ENZ therapy. Statistically significant differences (*p* < 0.05) were observed in cerebral infarction (1.27% vs. 1.83%) and peripheral vascular diseases (1.72% vs. 2.60%). Importantly, paroxysmal tachycardia (0.07%) was found only in the AA cohort. The other cardiovascular events showed no significant differences. Heart failure, followed by atrial fibrillation were the most common cardiovascular events for both cohorts. Hypotension in the AA cohort and peripheral vascular diseases in ENZ cohort were the third most common cardiovascular event outcomes for each group.

**Table 2:** Descriptive cardiovascular outcomes in the AA and ENZ cohorts.

Cardiovascular event outcomes	Abiraterone (*N* = 8,929)		Enzalutamide (*N* = 8,624)		*p*	STD
	*N*/Mean	%/STD	*N*/Mean	%/STD		
Any cardiovascular event	1,326	14.85	1321	15.32	0.3872	0.0131
Hypertension	16	0.18	10	0.12	0.2761	0.0164
Ischemic heart disease	17	0.19	16	0.13159	0.9407	0.0011
Myocardial infarction	21	0.24	19	0.22	0.8363	0.0031
Heart failure	432	4.84	415	4.81	0.9359	0.0012
Ventricular arrhythmias	139	1.56	146	1.69	0.4753	0.0108
Cerebral infarction	113	1.27	158	1.83	0.0023	0.0460
Peripheral vascular diseases	154	1.72	224	2.60	0.0001	0.0601
Atrial fibrillation	374	4.19	315	3.65	0.0675	0.0276
Paroxysmal tachycardia	6	0.07	0	0.00	0.0161	0.0364
Cardiomyopathy	43	0.48	48	0.56	0.4891	0.0104
Hypotension	256	2.87	218	2.53	0.1657	0.0209
Pulmonary embolism	125	1.40	101	1.17	0.1789	0.0203
Atherosclerosis	25	0.28	23	0.27	0.8662	0.0025
Aortic aneurysm	107	1.20	106	1.23	0.8523	0.0028

AA: abiraterone acetate; ENZ: enzalutamide; STD: standardised difference.

There were 7,647 patients matched in each cohort after controlling for age, socio-demographics, and comorbidity factors. When patients were matched, hyperlipidemia (21.50% vs. 20.87%) was the most common comorbidity in both cohorts, followed by diabetes (19.68% vs. 19.72%); the differences were not significant. Propensity score matching created similar samples in terms of demographic and clinical factors. Total healthcare expenditure, however, were slightly higher for the AA group even after matching ($22934.78 vs. $21862.99, *p* < 0.05) (Table 3).

**Table 3:** Baseline and outcomes characteristics of the study and comparison cohorts (Matched).

Variables	Abiraterone (*N* = 7,647)		Enzalutamide (*N* = 7,647)		*p*	STD
	*N*/Mean	%/STD	*N*/Mean	%/STD		
**Baseline characteristics**						
Age (years)	72.54	8.96	72.64	8.96	0.4685	0.0117
Age group						
65–74	3,020	39.49	2,995	39.17	0.7698	0.0067
75–84	2,363	30.90	2,342	30.63	0.7947	0.0059
85+	855	11.18	861	11.26	0.9134	0.0025
Comorbidity score						
CCI	2.22	1.14	2.22	1.13	0.8117	0.0038
CDS	2.22	2.57	2.23	2.64	0.7388	0.0054
Elixhauser Index	3.35	2.07	3.33	2.06	0.5602	0.0093
SES score by tertile						
Low	2,449	32.03	2,436	31.86	0.8733	0.0036
Medium	2,499	32.68	2,476	32.38	0.7789	0.0064
High	2,484	32.48	2,519	32.94	0.6697	0.0098
Comorbidities						
Depression	403	5.27	401	5.24	0.9591	0.0012
Diabetes	1,505	19.68	1,508	19.72	0.9656	0.0010
Gastric acid disorders	774	10.12	713	9.32	0.2391	0.0269
Hyperlipidemia	1,644	21.50	1,596	20.87	0.5018	0.0154
Osteoporosis	330	4.32	320	4.18	0.7768	0.0065
Asthma	178	2.33	176	2.30	0.9394	0.0017
COPD	406	5.31	422	5.52	0.6860	0.0092
Alzheimer’s disease	41	0.54	41	0.54	1.0000	0.0000
Pain syndrome	93	1.22	104	1.36	0.5770	0.0128
Health expenditures						
Total cost ($)	22934.78	23232.99	21862.99	22608.06	0.0038	0.0468
**Cardiovascular event outcome**						
Any cardiovascular event	1188	15.54	1134	14.83	0.3896	0.0197
Hypertension	12	0.16	10	0.13	0.7629	0.0069
Ischemic heart disease	16	0.21	12	0.16	0.5926	0.0122
Myocardial infarction	17	0.22	15	0.20	0.8024	0.0057
Heart failure	398	5.20	343	4.49	0.1430	0.0335
Ventricular arrhythmia	123	1.61	133	1.74	0.6558	0.0102
Cerebral infarction	106	1.39	140	1.83	0.1223	0.0353
Peripheral vascular disease	140	1.83	182	2.38	0.0944	0.0383
Atrial fibrillation	338	4.42	275	3.60	0.0663	0.0420
Paroxysmal tachycardia	4	0.05	0	0.00	0.1572	0.0324
Cardiomyopathy	38	0.50	40	0.52	0.8725	0.0037
Hypotension	224	2.93	190	2.48	0.2310	0.0274
Pulmonary embolism	111	1.45	92	1.20	0.3425	0.0217
Atherosclerosis	22	0.29	21	0.27	0.9140	0.0025
Aortic aneurysm	96	1.26	96	1.26	1.0000	0.0000

STD: standardised difference; SES: socioeconomic status; CCI: Charlson Comorbidity Index; CDS: Chronic Disease Score.

Table 4 demonstrates that, when demographic and clinical factors are controlled for at the baseline, there was no statistical difference in any cardiovascular event between the cohorts (15.54% vs. 14.83%). The most common cardiovascular event for both cohorts was heart failure (5.20% vs. 4.49%) followed by atrial fibrillation (4.42% vs. 3.60%), and hypotension (2.93% vs. 2.48%); again, the differences were not significant.

**Table 4:** Outcomes characteristics of the study and comparison cohorts (matched).

Cardiovascular event outcome	Abiraterone (*N* = 7647)		Enzalutamide (*N* = 7647)		*p*	STD
	*N*/Mean	%/STD	*N*/Mean	%/STD		
Any cardiovascular event	1188	15.54	1134	14.83	0.3896	0.0197
Hypertension	12	0.16	10	0.13	0.7629	0.0069
Ischemic heart disease	16	0.21	12	0.16	0.5926	0.0122
Myocardial infarction	17	0.22	15	0.20	0.8024	0.0057
Heart failure	398	5.20	343	4.49	0.1430	0.0335
Ventricular arrhythmias	123	1.61	133	1.74	0.6558	0.0102
Cerebral infarction	106	1.39	140	1.83	0.1223	0.0353
Peripheral vascular disease	140	1.83	182	2.38	0.0944	0.0383
Atrial fibrillation	338	4.42	275	3.60	0.0663	0.0420
Paroxysmal tachycardia	4	0.05	0	0.00	0.1572	0.0324
Cardiomyopathy	38	0.50	40	0.52	0.8725	0.0037
Hypotension	224	2.93	190	2.48	0.2310	0.0274
Pulmonary embolism	111	1.45	92	1.20	0.3425	0.0217
Atherosclerosis	22	0.29	21	0.27	0.9140	0.0025
Aortic aneurysm	96	1.26	96	1.26	1.0000	0.0000

STD: standardised difference.

## Discussion

Prostate cancer is the leading cause of cancer-related mortality, and its treatment often involves ADT such as AA and ENZ [3]. While these therapies have proven effective in managing mCRPC [3, 28] they come with a risk of adverse cardiovascular events [14, 28, 29]. Furthermore, the available literature on the treatment of metastatic prostate cancer with AA and ENZ predominantly comprises clinical trials, with a limited representation of real-world data. Clinical trials often exclude patients with significant comorbidities, posing a challenge in understanding the performance of these treatments in real-world scenarios. Our study showed that after controlling for age, SES, and sociodemographic and clinical factors, there was no significant difference in cardiovascular events between AA and ENZ, in agreement with the existing literature [15, 30, 31] Moreover, investigators such as George et al. have demonstrated that any difference in cardiovascular events for both AA and ENZ is minimal (hazard ratio, 1.23 vs. 1.10; *p* < 0.05) compared with the control [6].

Additionally, a comprehensive systematic review of real-world studies found few literature reviews on cardiovascular events [10]. Shah et al. found more pronounced cardiovascular events in patients using AA than ENZ therapy [10]; however, some of the articles identified included research populations with pre-existing cardiovascular conditions [10]. By contrast, we excluded patients with pre-existing CVD to distinguish new cases of CVD from ongoing episodes and to make a stronger correlation that the identified cardiovascular events were due to the adverse effects of the medication.

In our study, the median age for prostate cancer was 72 years old, and the most common prostate-specific comorbidity was diabetes for the ENZ group and hyperlipidemia for the AA group. When patients were matched, hyperlipidemia was the most common comorbidity in both cohorts, followed by diabetes. These results are consistent with previous literature [32–34]. Diabetes was found to be 28% higher in the ENZ cohort than in the AA cohort. Additionally, this supports our findings that the CCI and Elixhauser scores were higher in the ENZ group, indicating this cohort was sicker than the AA cohort.

Prostate cancer patients in higher-SES areas tended to use AA, whereas patients located in low-SES areas tended to use ENZ. The baseline health expenditure in the AA cohort was $1071.79 (*p <* 0.05) more than that in the ENZ cohort. Additional studies should be done to investigate the reason for these differences in SES and cost in depth, as they might be useful in understanding differences in patients’ prescribing patterns and help in better profiling the patients.

After controlling for age, sociodemographic characteristics, and clinical factors, we found that heart failure was the most common cardiovascular event, appearing at a 16% higher rate in the AA cohort than in the ENZ group. This aligns with previous studies that indicate the use of abiraterone was associated with a greater risk of cardiovascular-related hospitalisation compared to ENZ, for heart failure [16]. Furthermore, this aligns with prior research emphasising the need for vigilant monitoring of heart failure and CVD in these patients [35] Notably, only 4 patients in the AA cohort and no patients in the ENZ cohort had paroxysmal tachycardia as an adverse event. Moreover, compared with the ENZ cohort, nearly 23% more patients in the AA group developed atrial fibrillation. These findings have been discussed in previous literature where there is an elevated hazard ratio for arrhythmia-related hospitalisation in AA users than ENZ users [16]. This is further supported by an observational retrospective pharmacovigilance study, which found that AA was linked to a notably higher frequency of atrial tachyarrhythmia and heart failure compared with ENZ and other ADTs, likely due to AA’s tendency to induce hypermineralocorticism [16]. The increased risk of cardiovascular events associated with AA has been linked to its mineralocorticoid excess; to mitigate this adverse effect, it is indicated to prescribe it in combination with prednisone [16]. Therefore, patients predisposed to complications from heart failure, fluid overload, and arrhythmias should use AA with caution.

Although our study aligns with the individual cardiovascular findings for AA users, it is important to note that these findings were not statistically significant when controlled for sociodemographic and clinical factors. Furthermore, studies mentioned before like Hu, J. et al. comparative study between AA and ENZ included patients with pre-existing CVD which is different from our study population that excluded patients with pre-existing CVD and might account for the difference in their results with ours.

Notably, our study found hypotension as the third most common cardiovascular event in the AA group. This finding differentiates with preexisting literature that associates hypertension with AA and ENZ [7, 10, 36, 37]. A possible reason for this might be that hypertension was diagnosed using the ICD-10 code I15.9, which is used for secondary hypertension and is the most appropriate code for this scenario. Secondary hypertension is a type of hypertension that, by definition, is caused by an identifiable underlying primary cause (in this case, the use of medication AA and ENZ). However, primary hypertension accounts for 90%–95% of hypertension cases among adults, therefore it is prone to be coded more frequently in claims studies with its corresponding ICD-10 code, I10 [38]; this may account for the underdiagnosis of hypertension in our study [39]. Most frequent code used in claims studies is I10; therefore, we believe this might account for the underdiagnosis of hypertension in our study.

On the other hand, based on the pharmacovigilance study, patients who were administered ENZ had a lower incidence of cardiac events than those who were given AA [35]. While ENZ has been linked to hypertension [15], ischemic heart disease, and atrial fibrillation [40], our study specifically found a higher prevalence of peripheral vascular diseases and cerebrovascular disease among ENZ users. This aligns with previous research that indicates a greater association among ENZ users with peripheral vascular disease [10, 16]. Gupta et al. discuss how a few ADT studies have demonstrated to be associated with nonfatal cardiovascular events such as peripheral artery disease [14, 41, 42]. Although they do not establish whether peripheral artery disease is more common with ENZ, they state that there is an increased risk associated with ADT [14]. Additionally, although several meta-analyses have reported an increased risk of stroke in men with prostate cancer treated with ADT, they do not differentiate between AA and ENZ [14]. However, Jason Hu et al.’s study did suggest that ENZ users may be at greater risk of cerebrovascular stroke compared to ABI users, although this was also not statistically significant [16] This finding was very similar to our results. Furthermore, ENZ is known for its capacity to cross the blood-brain barrier and has also been associated with a multitude of adverse events relating to the CNS [16, 43]. It is important to mention, that several studies have mentioned the effect of ENZ in the CNS like seizures, fatigue, reduced cognitive function [44]; but few have looked at the effect on cerebrovascular disease. A call for further research on this is warranted.

## Limitations

This study has several limitations related to the use of administrative data sets, which may be subject to inaccurate coding of patient clinical diagnoses and procedures, as well as clinical information limited to conditions and treatments defined by ICD-10-CM codes and NDC codes. Since the analysis was done on the review of claims data that were not originally designed for research, some information is bound to be missing. Firstly, as with most claims-based data sources, there is a time lag between an individual’s receipt of services and when the files become available for research. The data may not be generalisable to the entire population, as some information may be missed in processing or reimbursement. Additionally, not all health data are captured in the claims. Both AA and ENZ have been proven to increase the survival of patients with metastatic hormone-sensitive disease naive to hormonal agents (i.e. a patient who has never used ADT therapy before); therefore, we focussed on patients who initiated treatment with these drugs to capture these populations [15]. We do recognise this can be a selection bias as we did not control for patients who might have used previous ADT therapy, which can be a limitation of our study.

It is important to note that although these medications might have had an effect on the incidence of CVD, we did control for this by excluding any patient who had previous CVD in the baseline period.

Findings of higher diabetes incidence in the ENZ cohort vs. the AA cohort could have been influenced by selection bias and should be interpreted with caution. Further, the absence of substantial differences between these drugs with regard to cardiovascular adverse events could be accounted for by improved treatment selection by physicians based on patient comorbidities which should be considered alongside these results.

Moreover, our study has potential limitations related to the use of area-based socioeconomic measures. Ideally, we would have been able to evaluate both individual and area-based socioeconomic factors, as relying solely on area-based measures can lead to misclassification of individuals across different socioeconomic strata within the same area. This misclassification occurs randomly, and its bias direction is known. If socioeconomic disparities exist within specific geographic regions, incorporating individual-level socioeconomic data would likely provide a more comprehensive understanding. Nonetheless, area-based measures offer valuable contextual information, taking into account various factors affecting all residents within an area, such as geographical location and the quality of public amenities such as hospitals. Our estimates would have been more precise if we had had access to more detailed individual-level socioeconomic data, allowing for a more nuanced examination of socioeconomic disparities.

## Conclusion

This study provides real-world evidence of the cardiovascular risk of prostate cancer patients treated with AA and ENZ that may not be apparent in clinical trial settings. After adjusting for prostate-specific comorbidities, age, SES, the likelihood of a cardiovascular event did not differ between AA and ENZ users. Our research may be of interest to clinicians treating patients with mCRPC, due to the high incidence of CVD in this population. This analysis adds to the discussion regarding the differences observed between AA and ENZ in terms of treatment-associated cardiovascular events and may aid clinicians when making informed treatment decisions in prescribing AA or ENZ.

## Data Availability

Data are not publicly available due to privacy and ethical restrictions.
